# Gene duplications in prokaryotes can be associated with environmental adaptation

**DOI:** 10.1186/1471-2164-11-588

**Published:** 2010-10-20

**Authors:** Marit S Bratlie, Jostein Johansen, Brad T Sherman, Da Wei Huang, Richard A Lempicki, Finn Drabløs

**Affiliations:** 1 Department of Cancer Research and Molecular Medicine, Norwegian University of Science and Technology, N-7006 Trondheim, Norway; 2 Laboratory of Immunopathogenesis and Bioinformatics, Clinical Services Program, SAIC-Frederick Inc., National Cancer Institute at Frederick, Frederick, MD 21702, USA

## Abstract

**Background:**

Gene duplication is a normal evolutionary process. If there is no selective advantage in keeping the duplicated gene, it is usually reduced to a pseudogene and disappears from the genome. However, some paralogs are retained. These gene products are likely to be beneficial to the organism, e.g. in adaptation to new environmental conditions. The aim of our analysis is to investigate the properties of paralog-forming genes in prokaryotes, and to analyse the role of these retained paralogs by relating gene properties to life style of the corresponding prokaryotes.

**Results:**

Paralogs were identified in a number of prokaryotes, and these paralogs were compared to singletons of persistent orthologs based on functional classification. This showed that the paralogs were associated with for example energy production, cell motility, ion transport, and defence mechanisms. A statistical overrepresentation analysis of gene and protein annotations was based on paralogs of the 200 prokaryotes with the highest fraction of paralog-forming genes. Biclustering of overrepresented gene ontology terms versus species was used to identify clusters of properties associated with clusters of species. The clusters were classified using similarity scores on properties and species to identify interesting clusters, and a subset of clusters were analysed by comparison to literature data. This analysis showed that paralogs often are associated with properties that are important for survival and proliferation of the specific organisms. This includes processes like ion transport, locomotion, chemotaxis and photosynthesis. However, the analysis also showed that the gene ontology terms sometimes were too general, imprecise or even misleading for automatic analysis.

**Conclusions:**

Properties described by gene ontology terms identified in the overrepresentation analysis are often consistent with individual prokaryote lifestyles and are likely to give a competitive advantage to the organism. Paralogs and singletons dominate different categories of functional classification, where paralogs in particular seem to be associated with processes involving interaction with the environment.

## Background

Orthologs and paralogs are two key concepts of evolutionary genomics. While orthologs are related via vertical descendent from a common ancestor, paralogs are related via duplication events subsequent to speciation [1]. For practical purposes paralogs are often defined as protein-coding sequences that have at least 30% sequence identity over more than 60% of their lengths [2,3]. In *Escherichia coli *K-12 as many as 30% of the proteins have at least one paralogous sequence in the genome [3]. The number of paralogs correlates well with genome size; a larger genome will in general have more paralogous genes [4].

According to Ohno [5], the first to both gather evidence for gene duplication and to describe the various fates of the duplicated genes, there are three possible outcomes of a duplication event where the gene duplicate is kept: neofunctionalization (the evolution of a new function in one of the duplicates), subfunctionalization (the division of ancestral functions among duplicates), and conservation of function (the conservation of all functions in both duplicates) [6]. If there is no selective advantage in keeping the duplicated gene, then the gene may become inactivated by mutations (nonfunctionalization), reduced to a pseudogene and finally removed from the genome by deletion. Actually only a small fraction of duplicated genes evolve new functions and are retained by the organism [7].

Expansion of genetic material represents an increased cost for most organisms. What is the evolutionary driving force behind retention of duplicated genes? New gene functions created by gene duplication may be a way of adapting to altered environments. The ability to adapt is crucial to the survival of the organism, and the duplicated genes may facilitate the handling of changed environmental conditions (e.g. nutritional scarcity or thermal stress) [8]. For the duplicated gene to avoid deletion, the gene must represent a positive response to environmental stress, e.g. by quickly picking up a mutation that makes the gene advantageous and selected for as a response to the new conditions, or by just increasing gene dosage as a response to higher demand [4]. When the selective pressure is removed, the paralogs may be lost again [8].

An alternative hypothesis of environmental adaptation has been proposed by Sanchez-Perez et al [9]. Instead of creating new gene functions by duplications this hypothesis implies that the gene copy performs the same function as the original gene, but that the paralogs function under different conditions. This kind of paralogs has been named ecoparalogs. An example of this process is seen in the hyperhalophilic bacterium *Salinibacter ruber*. This bacterium has halophilic proteins that have their optimal activity and stability at high salinity. Sanchez-Perez and his colleagues also found paralogous genes that performed the same function but differed in halophilicity; these genes could therefore function as backup genes to maintain essential functions over a wider range of salinity. Examples of ecoparalogs in other prokaryotes were also found, indicating that the presence of ecoparalogs in prokaryotic genomes could be frequent.

Prokaryotes can also acquire new genes and gene functions by horizontal gene transfer (HGT). HGT involves transfer of genes between different species, in contrast to the usual vertical inheritance. The gene transfer may happen between prokaryotes and eukaryotes, but it is more likely to happen between closely related organisms [10]. The transfer events can be classified into three different categories: acquisition of new genes, acquisition of genes similar to already existing genes (apparent paralogs) and xenologous gene displacement [11]. The latter involves displacement of a gene by a horizontally transferred ortholog from another lineage.

Gene duplication, gene loss and horizontal gene transfer are all considered to be important processes that shape prokaryotic genomes [8]. Although prokaryotic genomes are constantly changing because of these processes, the relative size of the genome is rather constant. Thus the two opposing factors of gene gain and gene loss are constantly balanced in prokaryotes. The ratio of genes per amount of DNA is found to be on average 1 kb per gene [12], indicating a relatively stable gene size and genome complexity.

In this analysis we have identified annotation terms that are statistically overrepresented in paranomes (set of paralogs) of all fully sequenced prokaryotes at the time of this study, and then performed a biclustering of properties and species based on overrepresented Gene Ontology (GO) terms.

Statistical overrepresentation analysis is a well established method for finding significantly overrepresented features in a data set, commonly used e.g. for analysing gene sets [13] or transcription factor binding sites [14]. The data set is compared against a background (reference) set representing the average or typical gene set. For each term the number of objects with and without that term in the data set and in the background set is counted, creating a 2 × 2 matrix, and the statistical significance of the distribution is tested using e.g. Fisher's exact test.

GO was created as a tool to get a more unified and standard description of genes and their functions in eukaryotes and prokaryotes, in particular since it was found that a large fraction of genes specifying the core biological functions are shared among species. Gene products are described in terms of biological process, cellular components and molecular functions, independent of species [15]. The GO network has a tree structure, where each node (GO term) is the child of a parent, and each child may have more than one parent. Each GO term has a unique GO identifier.

Biclustering as it is implemented e.g. in the Biclustering Analysis Toolbox (BicAT) [16] clusters a data set in two dimensions simultaneously. This makes it possible to automatically identify clusters of organisms based on similarity within different subsets (clusters) of features, giving both an overview of features (GO terms) that define individual clusters as well as organisms that are associated with these clusters. Biclustering, or co-clustering, has become a popular way of analysing e.g. gene expression data. The technique may define several alternative solutions of partly overlapping clusters because it is possible for an organism - GO pair to participate in more than one cluster, which could not be achieved by more traditional clustering.

Identification of overrepresented features of the paranomes in a large number of prokaryotes has made it possible to analyse selected features to assess the hypothesis that they may reflect how the prokaryotes interact with the environment, e.g. through gene transfer, locomotion, chemotaxis, ion transport or photosynthesis.

## Results and discussion

**Figure 1:**
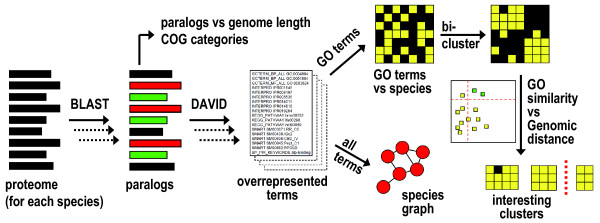
**General data flow in the analysis**. For each organism the proteome was extracted and paralogs were identified with Blast searching. The resulting paranomes were analysed by plotting the number of genes with paralogs vs. genome length (Figure 2) and the distribution of COG categories (Figure 3). The paranomes were analysed for statistical overrepresentation of annotation terms using DAVID. The output from DAVID was initially analysed as a graph (Figure 4), using overlap between complete annotation lists from DAVID to define pairwise similarities between species. Clusters in the graph were identified with visual inspection (Table 1). For a more stringent analysis only GO terms were used, and these were analysed with biclustering using a matrix consisting of individual GO term occurrences for each species. First data based on three different background models for DAVID were compared (Figure 5). Clusters for the optimal background model were then analysed for GO terms frequently associated with specific species (Table 2). Interesting clusters in the biclustering were identified by analysing GO similarity versus genomic distance (Figure 6), and selected clusters representing simultaneously both high GO similarity and large evolutionary distance (Table 3) were discussed in relationship to literature data.

The general data flow of the analysis is illustrated in Figure 1, starting with the full proteome of each prokaryote. The main analysis consists of mapping of paralogs, identification of statistically overrepresented annotation terms and biclustering of annotation terms versus species in order to identify potentially interesting clusters. See the figure legend for a more detailed description.

### Paralogs were identified with focus on recent duplications

We identified paralogs in 897 prokaryotes by sequence analysis, using all fully sequenced prokaryotic genomes (archaea and bacteria) from NCBI [17] at the time of analysis. The 200 prokaryotes with the highest paralog fraction were included in the full analysis (Table S1 in Additional File 1), see Methods. Among these organisms were 10 archaea. The paralog fraction was defined as the ratio between the number of proteins with one or more paralogs (not considering the number of copies) and the total number of proteins in that particular proteome, and is also known as the degree of duplication. The organism with highest paralog fraction in our analyses was *Aster yellows witches-broom phytoplasma *(strain AYWB) (12.12%), the organism with lowest paralog fraction (among the top 200 organisms) was *Streptococcus pyogenes *(strain MGAS8232) (2.28%).

Our paralog criterion was set to 75% identity between sequences. This is a fairly strict cut-off, as a more commonly used criterion is 30% sequence identity over more than 60% of the sequence length. By setting a relatively strict sequence identity cut-off, our analyses will focus on recently arisen or well conserved paralogs. The paralogs are therefore likely to have retained the same or at least a very similar function, so that they represent a real and often recent amplification of this particular function. In this way we are focusing on functions where the prokaryote may have had a recent need to increase the gene dosage, e.g. to adapt to a changing environment or new niche in which it probably is living right now. However, the strict cut-off makes it difficult to compare the paralog fraction in our analysis with other analyses where a less strict criterion has been used.

It is possible that some of the genes in our analyses are not "real" paralogs, as they may have been acquired by horizontal gene transfer. Horizontally transferred genes from species that are not closely related to the host will in most cases be less similar to the copy in the recipient genome. Our strict paralog cut-off therefore makes it likely that genes from horizontal gene transfer constitute a very small part of the data set, unless they in fact are true paralogs that have been copied into the genome more than once.

### Number of paralogs is correlated with genome size

**Figure 2:**
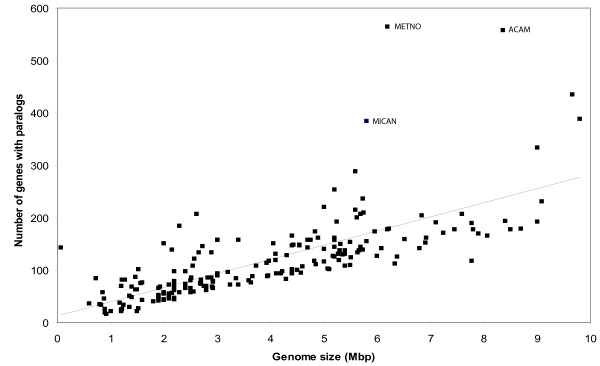
**Number of genes with paralogs versus genome size**. Relationship between numbers of genes having paralogs versus genome size for 200 prokaryotes, including trend line (trend line equation y = 26.99x + 13.40 where y and x stand for "number of genes with paralogs" and "genome size" respectively, correlation coefficient r = 0.73). In particular three genomes differ from the linear trend; these are *M. aeruginosa *(MICAN), *M. nodulans *(METNO), and *A. marina *(ACAM). When these outliers are excluded, correlation coefficient r = 0.80 (trend line equation y = 23.89x + 20.59).

Earlier analyses [18,19] have shown that there is a linear correlation between the number of paralogs and the genome size of an organism. To confirm this relationship, we plotted the number of genes with paralogs (not considering the number of copies) versus genome size, as shown in Figure 2. The figure shows that there is a relatively good correlation (r = 0.73 when outliers are included, r = 0.80 when outliers are excluded) between genome size and the number of genes with paralogs. However, three genomes (indicated in the figure) have clearly more paralogs compared to the general trend; these are *Microcystis aeruginosa*, *Methylobacterium nodulans*, and *Acaryochloris marina*. The genome of the cyanobacterium *M. aeruginosa *is known to have high plasticity, and as much as 11.8% of the genome consists of insertion sequences and transposable elements [20]. This may explain the high number of genes with paralogs. The alphaproteobacterium *M. nodulans *and cyanobacterium *A. marina *have a total of 7 and 9 plasmids each [21]. Only the size of the main chromosome was used for the figure, thus these two genomes appear smaller than they actually are.

### Distribution of functional categories differs between paralogs and singletons

**Figure 3:**
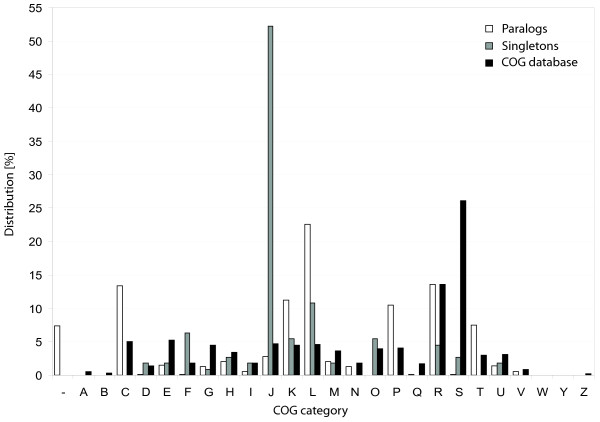
**Distribution of COG categories**. The figure shows distribution of paralogs and persistent singletons according to COG categories, as well as the general distribution of the COG database. '-' is used where no COG group could be identified. The different categories are A: RNA processing and modification, B: chromatin structure and dynamics, C: energy production and conversion, D: cell-cycle control and mitosis, E: amino acid metabolism and transport, F: nucleotide metabolism and transport, G: carbohydrate metabolism and transport, H: coenzyme metabolism, I: lipid metabolism, J: translation, K: transcription, L: replication and repair, M: cell wall/membrane/envelope biogenesis, N: cell motility, O: post-translational modification, protein turnover, chaperone functions, P: inorganic ion transport and metabolism, Q: secondary metabolites biosynthesis, transport and catabolism, R: general functional prediction only, S: function unknown, T: signal transduction, U: intracellular trafficking and secretion, V: defence mechanisms, W: extracellular structures, Y: nuclear structure, Z: cytoskeleton. COG classification A, Y, and Z are not used for prokaryotic COGs.

The functional classification of paralog-forming genes was compared to paralog-less genes (singletons) that are conserved in the majority of prokaryotic genomes, so-called persistent singletons. The paralogs were classified according to function by using the Clusters of Orthologous Groups (COG) classification in the protein table files from NCBI [22], further details are given in Methods. The singletons data were extracted from a published data set [23]. The paralog and singleton sets were compared to the full set of entries in the COG database [24], see Figure 3.

It is difficult to compare the distribution of paralogs and singletons directly, as the ratios are sensitive e.g. to whether ribosomal proteins are included or not. We therefore focussed mainly on COG classes where either paralogs or singletons are close to zero, thereby reducing the importance of the relative scaling. This showed that paralogs are preferentially associated with energy production and conversion (COG category C), cell motility (N), inorganic ion transport and metabolism (P), signal transduction (T), and defence mechanisms (V). The singletons are preferentially associated with cell-cycle control and mitosis (D), nucleotide metabolism and transport (F), and post-translational modification (O), indicating that these processes are under more strict control, not allowing for duplications. For both groups replication and repair (L) is important, and to some extent transcription (K).

The features that describe the set of paralogs are features that may contribute to a competitive advantage for the organism. By having more copies of genes in the category inorganic ion transport and metabolism the organisms may enhance the uptake of trace metals that are important for survival. The accessibility of such essential trace metals (e.g. copper and iron) in some environments may be scarce and difficult to utilize [25], and enhancing the functional capabilities may be of great advantage. The COG category for defence mechanisms is also associated with paralogs. Several bacteria live in environments that are stressful for the organism, e.g. environments with harmful contaminations, lack of nutrition or environments with a high or low temperature. All of these factors may be stressful and cause damage to DNA. Therefore, by having more copies of replication/repair genes, the organism adds extra robustness to its repair system. Extra copies of genes involved in cell motility may also be advantageous, making it possible to move around in more variable environments.

A similar analysis has been performed by Pushker et al [19] where they analysed the differences of functional classification between large gene families with more than five members (paralogs) and singletons in *E. coli *K-12 and *Bacillus subtilis*. For *E. coli *they found that genes involved in transport of metabolites were overrepresented, followed by genes for transcription and replication/repair. A high association of paralogs with amino acid metabolism was also confirmed by an analysis performed by Gevers et al [8]; they assigned functional classes to genes of 48 genomes. In addition, they found retained duplicates in COG categories transcription (K) and inorganic ion metabolism (P) and to a lesser extent in carbohydrate metabolism (G), defence mechanisms (V), and energy production and conversion (C). Our analyses confirm most of these results, in particular regarding inorganic ions, defence mechanisms and energy production, but we found a quite low distribution of paralogs involved in transport of metabolites. The differences in functional classification may to some extent be caused by our strict paralog criterion, which may rule out paralogs that have adapted to new functions, e.g. transport of alternative metabolites.

Pushker et al also found that singletons were almost equally distributed among the functional categories (excluding genes with an unknown function). This is different from our analysis, where singletons in particular are overrepresented in the translation category (J). This category consists mainly of ribosomal proteins, and those genes are usually found with only one copy in the genome [23]. The singletons discussed here are so-called persistent singletons; i.e. they are found in the majority of the genomes included in the analysis. The singletons in the analysis of Pushker et al are not persistent, and the handling of ribosomal proteins is not described in their analysis. The two analyses are therefore not directly comparable.

### Prokaryotes can be clustered based on shared features

**Figure 4:**
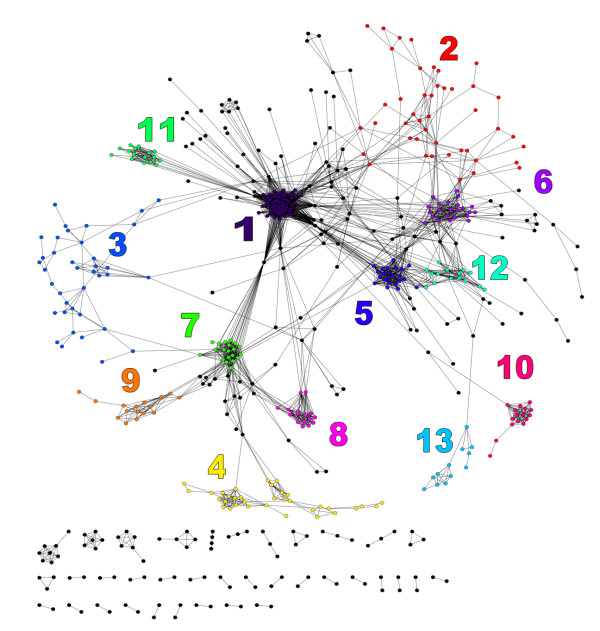
**Graph-representation of similarity between species**. The graph shows individual organisms as nodes, and the edges represent strong overlap between annotation lists from DAVID (See Methods for details). Layout was computed by Cytoscape. Clusters were selected by visual inspection, and are numbered according to Table 1.

We used the Database for Annotation and Visualization and Integrated Discovery (DAVID) [26] and associated tools to identify statistically overrepresented annotation terms for the set of paralogs from each prokaryote. To get an initial overview of the full data set we did a pairwise comparison of all species by matching the lists of overrepresented terms and counting the number of terms that were found in both lists. The final list of pairwise similarities between species was then used as input to Cytoscape [27], using species (i.e. paranomes) as nodes and the number of shared annotation terms as edge weights. An automatic layout approach was used for the resulting graph (Figure 4), and a selection of potentially interesting clusters was identified by visual inspection of this graph (Table 1).

**Table 1:** Clusters from the graph visualization of species

Clus	N	Main species	Main terms
1	65	Shigella sp. (10.8%) Yersinia pestis (7.7%)	GO BP 0006259 DNA metabolic process (98.5%) GO BP 0006313 transposition, DNA-mediated (75.8%)
2	44	Burkholderia sp. (15.9%)	GO BP 0006810 transport (37.8%)
3	39	Pseudomonas sp. (25.6%) Methanococcus sp. (15.4%)	GO BP 0006082 organic acid metabolic process (37.5%) GO BP 0008652 cellular amino acid biosynthetic process (35.0%)
4	34	Streptococcus sp. (47.1%) Clostridium sp. (20.6%)	GO BP 0005975 carbohydrate metabolic process (40.0%) GO MF 0050044 galactose-6-phosphate isomerase activity (31.4%)
5	23	Synechococcus sp. (30.4%) Prochlorococcus marinus (26.1%)	GO BP 0015979 photosynthesis (91.7%)
6	23	Burkholderia sp. (13.0%)	GO BP 0045449 regulation of transcription (91.7%)
7	19	Escherichia coli (89.5%)	GO MF 0051539 4 iron, 4 sulfur cluster binding (90.0%)
8	16	Salmonella enterica (87.5%)	GO BP 0017004 cytochrome complex assembly (94.1%) GO MF 0016829 lyase activity (88.2%)
9	16	Bacillus sp. (87.5%)	GO MF 0022857 transmembrane transporter activity (64.7%) GO BP 0009254 peptidoglycan turnover (64.7%)
10	16	Staphylococcus sp. (100%)	GO CC 0005576 extracellular region (82.4%) GO BP 0009405 pathogenesis (76.5%)
11	16	Campylobacter jejuni (18.8%)	GO CC 0044460 flagellum part (88.2%)
12	15	Rhodopseudomonas palustris (33.3%)	GO BP 0040011 locomotion (93.8%) GO BP 0006935 chemotaxis (93.8%)
13	12	Mycobacterium sp. (83.3%)	INTERPRO IPR009416 Protein of unknown function (76.9%) COG ONTOLOGY Cell motility and secretion (46.2%)

For each cluster (Clus) in Figure 4 the number of species (N), the dominating species (in percentage of N) and selected dominating annotation term(s) from the overrepresentation analysis (in percentage of N in which this term was found) is shown. Several annotation terms will be highly similar, representing the same type of property, therefore only a selection of terms is shown.

The resulting clusters gave an interesting overview of the data set. The graph itself shows some clear clusters, but also a high level of connectivity (edges) between clusters, even though a quite strict criterion has been used. The overlap was required to consist of at least 10 annotation terms, and it should cover at least 30% of the maximum possible overlap (see Figure S1 in Additional File 2 and Methods). This gave a network of 567 nodes and 3796 edges, whereas an overlap of at least 3 annotation terms without any requirement on relative size of the overlap gave a network consisting of 826 nodes and 29755 edges. The latter network illustrates the extensive connectivity in the data set, although it is too complex for identification of clusters. This shows that the data set can (and probably should) be clustered in several different ways.

Some clusters in Table 1 are dominated by multiple strains of a single species, e.g. cluster 7 (*Escherichia coli*), or by closely related species, e.g. clusters 9 (*Bacillus *sp.), 10 (*Staphylococcus *sp.), and 13 (*Mycobacterium *sp.). More interesting are clusters with high diversity with respect to species, but low diversity with respect to function, e.g. clusters 1 (transposition), 5 (photosynthesis), 6 (regulation of transcription), 11 (flagellum), and 12 (locomotion, chemotaxis). Most of these seem to be associated with how the prokaryotes interact with and respond to changes in the environment.

This analysis gave a useful overview of the data set. However, the analysis is obviously sensitive to several factors like annotation level, similarity cut-off, and graph layout. It was therefore relevant to use a more well-defined approach for this data set, focusing on the 200 species with largest paralog fraction, using a high-quality subset of annotation terms and flexible clustering.

### Using all paralogs as background gives the most homogenous clusters

We started by analysing how the choice of background set for overrepresentation analysis would influence the clustering result, using three different background sets. The test set consisting of the paranome for each organism was the same in all cases, whereas the background sets either were all paralogs (i.e. the full paranome), all proteomes, or the individual proteome for each organism. The overrepresentation analysis performed by DAVID resulted in annotation terms within a range of different categories, e.g. InterPro, SMART, PIR SuperFamily, KEGG, COG, and GO. The latter was the best represented category; therefore GO [15] was best suited to describe features of the paranomes. The overrepresented GO terms for the paralogs in our test set were therefore used for analysis.

The subsequent data analysis was going to be based on biclustering on GO terms and species. It is a reasonable hypothesis that the best data set for clustering is the one forming the most homogenous clusters with respect to GO, so that the clusters tend to contain closely related GO terms. This favours clusters representing a single dominating feature. We used BicAT [16] with the BiMax algorithm on the test set versus each of the three background sets (full results from BicAT are not shown, but are available upon request). The clustering from BicAT using all paralogs as background resulted in 1534 clusters and included 167 organisms; meaning that 33 of the 200 initial organisms were not included. The majority of these (29) contained none or only one GO term and could therefore not be clustered by BicAT using GO terms only. There were also 9 organisms with only two GO terms. The combination of the two GO terms had to be found in at least one other organism for these organisms to be included in any clusters. This was not the case for four organisms; therefore only 167 genomes could be clustered. We also chose to remove clusters containing only different strains of *E. coli *(~110 clusters, or 7%), as the high number of closely related strains would cause large and artificially uniform clusters.

**Figure 5:**
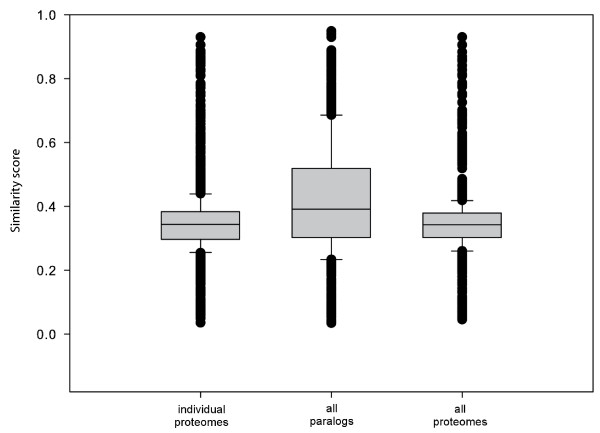
**Within-cluster GO similarities for alternative background models**. The distribution of within-cluster similarity scores for GO terms computed over all clusters from DAVID using three different background models: individual proteomes, all paralogs, and all proteomes. A higher score implies a more similar or unambiguous cluster with respect to GO terms, which is what we try to achieve to facilitate the subsequent analysis of clusters. The average for all paralogs is significantly different from the average for individual proteomes and all proteomes (p = 2.01 × 10^-47 ^and p = 1.95 × 10^-65 ^respectively using a two-sided t-test).

We then used Gene Semantic Similarity Analysis and Measurement Tools (G-SESAME) [28] to measure the similarity of GO terms within each cluster. This program encodes the biological meaning of GO terms into a numeric value by aggregating the semantic contributions of their ancestor terms [28]. The clusters get a score value between 0 and 1; a higher score implies a more similar or unambiguous cluster. The similarity score for each of the three analyses is shown in Figure 5. From this figure we see that clusters from the overrepresentation analysis using the background set consisting of all paralogs got the highest average similarity score, thus implying the most unambiguous clusters.

This result seems reasonable based on the properties of these background sets. Using the full set of proteomes as background will highlight how the paralogs are different from the complete set of proteins. However, this will include a large number of proteins that never are involved in any comparable processes, and because of that almost any combination of paralogs may seem to be somehow unique, compared to the more inflated background set. The same will obviously be true when we use each individual proteome as background. This will still contain a large number of genes involved in other processes. However, if we use the full paranome as background we highlight how the evolutionary process of a given organism is unique compared to evolutionary processes in general. This is a far more relevant question, which highlights unique features of individual organisms. This is also consistent with the more general guideline for overrepresentation analysis, "to set up the population background as the pool of genes that have a chance to be selected for the studied annotation category in the scope of the users' particular study" [29].

The set of overrepresented GO terms identified using the full paranome as background was therefore chosen for further analyses. The phrase "overrepresented GO terms" will be used to denote the GO terms retrieved from the DAVID analysis of this data set. The BicAT clustering output gave several partly overlapping clusters, representing alternative solutions to the biclustering problem. First we identified frequent co-occurrences of species and features (GO terms) in these clusters, as this probably represents features that are strongly associated with individual species. Next we identified clusters showing indications of convergent evolution of species with respect to common features (see below).

### The frequency of GO associations can be related to properties of the organism

To get an overview of frequent associations between GO terms and species we counted all GO term - species co-occurrences over all BicAT clusters. The frequency of each association indicates the importance of the GO term in defining a relevant property for that species. The results are presented in Additional file 3 as Table S2. The GO terms are listed according to which organism they are associated with, making it easy to identify important features for a given species. However, many highly similar strains of the same species will give an artificially strong species - GO term association; therefore the 11 strains of *E. coli *originally included in the data set were removed from this analysis.

From the complete table (Table S2 in Additional file 3) we see that the GO terms most frequently associated with a given species in biclustering are rather general. Some combinations appear quite often, in the range of 40-60 times, and typical GO terms are regulation of macromolecule metabolic process (GO:0060255), biological regulation (GO:0065007), macromolecule metabolic process (GO:0043170), DNA metabolic process (GO:0006259), biological regulation (GO:0065007), ribonucleotide binding (GO:0032553), purine nucleoside binding (GO:0001883), intracellular organelle part (GO:0044446), glycosaminoglycan catabolic process (GO:0006027), and polysaccharide metabolic process (GO:0005976). These terms are in most cases too general, making it difficult to identify any specific association between lifestyle and paralogs. To be able to draw any reliable conclusions regarding important features associated with individual species we have chosen to base the discussion on a subset. Table 2 presents a representative subset of Table S2, focusing on GO terms representing specific processes or functions, rather than more general processes.

**Table 2:** Frequent species - GO terms associations

Bacteroides thetaiotaomicron VPI-5482 (Bacteroidetes/Chlorobi)		
GO:0005976	26	polysaccharide metabolic process
GO:0005975	21	carbohydrate metabolic process
GO:0044275	21	cellular carbohydrate catabolic process
GO:0000272	20	polysaccharide catabolic process
GO:0006027	20	glycosaminoglycan catabolic process
GO:0009253	20	peptidoglycan catabolic process
GO:0016052	18	carbohydrate catabolic process
GO:0044265	16	cellular macromolecule catabolic process
GO:0044262	15	cellular carbohydrate metabolic process
GO:0030312	11	external encapsulating structure
GO:0009279	7	cell outer membrane
GO:0031975	7	envelope
GO:0016301	5	kinase activity
GO:0016811	5	hydrolase activity, acting on carbon-nitrogen (but not peptide) bonds, in linear amides
GO:0016853	5	isomerase activity
GO:0019867	5	outer membrane
GO:0030313	5	cell envelope
GO:0044462	5	external encapsulating structure part
GO:0004871	4	signal transducer activity
GO:0060089	4	molecular transducer activity
GO:0016020	3	membrane
GO:0008643	2	carbohydrate transport

**Methylibium petroleiphilum PM1 (Betaproteobacteria)**		

GO:0051186	5	cofactor metabolic process
GO:0006766	4	vitamin metabolic process
GO:0006767	4	water-soluble vitamin metabolic process
GO:0046483	4	heterocycle metabolic process
GO:0009235	3	cobalamin metabolic process
GO:0009236	3	cobalamin biosynthetic process
GO:0006778	2	porphyrin metabolic process
GO:0006779	2	porphyrin biosynthetic process
GO:0009110	2	vitamin biosynthetic process
GO:0033013	2	tetrapyrrole metabolic process
GO:0033014	2	tetrapyrrole biosynthetic process
GO:0042364	2	water-soluble vitamin biosynthetic process
GO:0051188	2	cofactor biosynthetic process

**Nitrobacter hamburgensis X14 (Alphaproteobacteria)**		

GO:0043167	10	ion binding
GO:0043169	10	cation binding
GO:0046872	10	metal ion binding
GO:0046914	8	transition metal ion binding
GO:0042126	6	nitrate metabolic process
GO:0005507	5	copper ion binding
GO:0008940	5	nitrate reductase activity
GO:0009325	5	nitrate reductase complex
GO:0016661	4	oxidoreductase activity, acting on other nitrogenous compounds as donors
GO:0004129	3	cytochrome-c oxidase activity
GO:0015002	3	heme-copper terminal oxidase activity
GO:0016675	3	oxidoreductase activity, acting on heme group of donors
GO:0016676	3	oxidoreductase activity, acting on heme group of donors, oxygen as acceptor
GO:0020037	3	heme binding

**Nostoc punctiforme PCC 73102 (Cyanobacteria)**		

GO:0015979	11	photosynthesis
GO:0030076	9	light-harvesting complex
GO:0042716	9	plasma membrane-derived chromatophore
GO:0031410	8	cytoplasmic vesicle
GO:0031982	8	vesicle
GO:0032991	8	macromolecular complex
GO:0044433	7	cytoplasmic vesicle part
GO:0019684	5	photosynthesis, light reaction
GO:0043234	4	protein complex
GO:0009767	3	photosynthetic electron transport chain
GO:0009772	3	photosynthetic electron transport in photosystem II
GO:0030077	3	plasma membrane light-harvesting complex

**Polaromonas naphthalenivorans CJ2 (Betaproteobacteria)**		

GO:0006281	5	DNA repair
GO:0006974	5	response to DNA damage stimulus
GO:0034984	5	cellular response to DNA damage stimulus
GO:0033554	4	cellular response to stress
GO:0051716	4	cellular response to stimulus
GO:0006950	2	response to stress
GO:0043565	2	sequence-specific DNA binding

A subset of five organisms from the analysed data set, showing the frequency of GO terms associated with these organisms over all clusters. Phylogenetic class of each organism is indicated.

For *Bacteroides thetaiotaomicron *the GO terms concerning polysaccharides and membrane are overrepresented. This is in accordance with the genome annotation done by Xu et al [30], as they found genes involved in polysaccharide uptake and degradation to be one of the most markedly expanded paralogous groups. *B. thetaiotaomicron *belongs to the group of Bacteroides and is found in the human colon [31]. The ability to utilize otherwise indigestible polysaccharides, often referred to as "dietary fibre", is probably one of the reasons that Bacteroides are one of the predominant genera in the colon [32]. The genome of *B. thetaiotaomicron *also encodes many outer membrane proteins that are likely to be involved in acquisition of polysaccharides [30]. *Methylibium petroleiphilum *belongs to the betaproteobacteria and has the capability to metabolize the fuel oxygenate methyl *tert*-butyl ether (MTBE) [33], a persistent groundwater contaminant [34]. It also degrades aromatic and straight-chain hydrocarbons found in petroleum products [33]. Overrepresented GO terms are cobalamin metabolic process, tetrapyrrole metabolic process, and cofactor metabolic process. A study by Rohwerder et al [35] investigated the degradation pathway of 2-hydroxyisobutyric acid (2-HIBA), an intermediate in the degradation pathway of MTBE. The study was performed with the MTBE-degrading betaproteobacteria-strain L108. The results showed a cobalt and cobalamin dependence for degradation of MTBE. When growing in a cobalt-deficient medium, degradation rate and growth rate were significantly reduced [35]. Oxidoreductase activity is one of the overrepresented GO terms for *Nitrobacter hamburgensis*. The bacterium gains energy by oxidation of nitrite to nitrate [36], thus the retained duplicates are likely to enhance the energy conservation for this organism. *Nostoc punctiforme *is a member of the genus Cyanobacteria, and GO terms for photosynthesis and vesicles are overrepresented. Cyanobacteria are photosynthetic bacteria that carry out photosynthesis [37], this result is also supported by hits in the KEGG pathway for photosynthesis (npu00195) in the overrepresentation analysis retrieved from DAVID. *Polaromonas naphthalenivorans *(strain CJ2) is known to metabolize naphthalene *in situ *[38]. We find GO terms regarding DNA repair, DNA damage and stress response as overrepresented. Earlier analyses have shown evidence that naphthalene damages DNA, membrane, and tissue [39], which is likely to explain the numerous paralogs associated with these GO terms.

The subset of data presented in Table 2 illustrates that key paralogs in a species are indeed related to important features of the organism, giving it a likely advantage in the competition for survival. However, in the complete table (Table S2 in Additional file 3) there are also features or functions where the link to survival is less obvious. *Bartonella tribocorum *(strain CIP 105476), *Magnetococcus sp*. (strain MC-1), *Onion yellows (OY) phytoplasma*, *Orientia tsutsugamushi *(strain Boryong and Ikeda), and *Paracoccus denitrificans *(strain PD1222) are often associated with GO terms for DNA methylation and alkylation. Earlier analyses have shown that DNA methylation may be a versatile regulator of virulence expression and that DNA adenine methyltransferase may have an effect on invasion into and adhesion to host cells of some but not all pathogens [40]. Methylation and the level of DNA adenine methyltransferase may also influence gene transcription, DNA mismatch repair, chromosome replication initiation, and nucleoid structure [41]. *B. tribocorum*, *OY phytoplasma*, and *O. tsutsugamushi *are known pathogens, while *Magnetococcus sp*. and *P. denitrificans *are not. For the pathogenic bacteria, these paralogs may therefore play a role in pathogenicity, while the reason for multiple copies of methyltransferase in the other bacteria remains unknown. A similar result was the frequent combination of GO terms for nucleus (GO:0005634) and nuclear part (GO:0044428) with *Halobacterium salinarum *(strain R1), *Halobacterium sp., Halorubrum lacusprofundi *(strain ATCC 49239), *Natronomonas pharaonis, M. aeruginosa *(strain NIES 843), and *Photorhabdus luminescens*. Since prokaryotes do not have a nucleus, this is an unexpected result. Most likely, this is a consequence of some of the paralogs in our data set having orthologs that are involved in reactions regarding nucleus in eukaryotes. This highlights an important limitation when using GO terms in automatic data analysis.

### Clusters with high GO similarity and large phylogenetic distances can highlight common environmental factors

A second approach to analysing the biclustering data focused on identification of possible convergent evolution. The trivial explanation for high similarity of GO terms within a cluster is of course that the genomes are closely related. It is not possible to link such similarity to adaptive processes. However, by taking the phylogenetic distances within a cluster into account we can identify clusters with high similarity of GO terms despite non-similar genomes. In other words, if a cluster from biclustering has a high internal similarity score on GO terms and a large phylogenetic distance, it is possible that there are processes stimulating some level of convergent evolution for organisms in that particular cluster.

**Figure 6:**
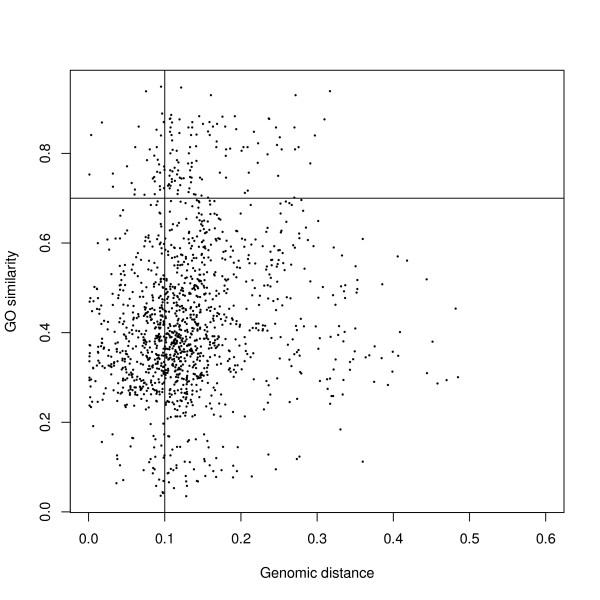
**Functional similarity (GO) and phylogenetic distance for all clusters**. Each point represents a cluster from biclustering with similarity score over GO terms (G-SESAME) and phylogenetic distance between genomes (Kr-algorithm). Cut-off values used for identification of interesting clusters with high GO similarity and large genomic distance are indicated.

A distance matrix for all the organisms in our analyses were computed using the Kr-algorithm [42] with 16S ribosomal protein sequences as input, and by combining this information with the G-SESAME distance matrix on GO terms we were able to identify clusters with a high internal similarity score and a large phylogenetic distance. The Kr-algorithm computes distances between unaligned DNA sequences, and this is advantageous for distantly related species. The evolutionary distances estimated by the Kr-algorithm were verified by comparison to a pre-computed distance matrix from the Ribosomal Database Project (RDP) [43]. Further details on this verification are given in Methods. Figure 6 shows the distribution of GO similarity scores and phylogenetic distances for all clusters. To sort out the interesting clusters, we defined a cut-off at > 0.1 for genomic distance and > 0.7 for GO similarity (see Figure 6). These values were estimated to be a reasonable compromise between genomic diversity, GO similarity and number of clusters returned for further analysis. This resulted in a list of 104 clusters, most of these clusters contained relatively few or rather general GO terms like catabolic process, intracellular components, membrane, biosynthetic process, and nuclease activity. A list of these clusters is given as Additional file 4, Table S3. However, there were some clusters with more specialized GO terms, and a representative subset of these is given in Table 3, focusing on species where relevant literature data could be found. In case of overlapping clusters, the cluster with fewest organisms was included in the table. The six clusters may be briefly summarized by the descriptions metal ion binding, cell motility, glycoside hydrolase, methyltransferase, ion transport, and GTPase domain, indicating that at least some of these clusters represent interaction with the environment.

**Table 3:** A representative subset of 6 clusters with relatively specific GO terms

Cluster 1 - COG classification C (Energy production and conversion)			
**GO terms**	GO:0005507	copper ion binding	function
	GO:0043167	ion binding	function
	GO:0043169	cation binding	function
	GO:0046872	metal ion binding	function
	GO:0046914	transition metal ion binding	function
			
**Organisms**	Dinoroseobacter_shibae_DFL_12	bacteria	Alphaprot.
	Mycobacterium_gilvum_PYR-GCK	bacteria	Actinobac.
	Nitrobacter_hamburgensis_X14	bacteria	Alphaprot.
			

**Cluster 2 - COG classification N (Cell motility and secretion)**			

**GO terms**	GO:0009288	bacterial-type flagellum	component
	GO:0019861	flagellum	component
	GO:0042995	cell projection	component
	GO:0044460	flagellum part	component
	GO:0044461	bacterial-type flagellum part	component
	GO:0044463	cell projection part	component
			
**Organisms**	Alkaliphilus_metalliredigens_QYMF	bacteria	Firmicutes
	Desulfovibrio_desulfuricans_G20	bacteria	Deltaprot.
	Magnetococcus_MC-1	bacteria	Other Bacteria
	Xanthomonas_oryzae_PXO99A	bacteria	Gammaprot.
			

**Cluster 3 - COG classification R (General function prediction only)**			

**GO terms**	GO:0004553	hydrolase activity, hydrolyzing O-glycosyl compounds	function
	GO:0016798	hydrolase activity, acting on glycosyl bonds	function
			
**Organisms**	Anaerocellum_thermophilum_DSM_6725	bacteria	Firmicutes
	Escherichia_coli_O157_H7_EC4115	bacteria	Gammaprot.
	Escherichia_coli_O157H7	bacteria	Gammaprot.
	Sodalis_glossinidius_morsitans	bacteria	Gammaprot.
	Streptococcus_pyogenes_MGAS315	bacteria	Firmicutes
	Streptococcus_pyogenes_SSI-1	bacteria	Firmicutes
	Streptomyces_coelicolor	bacteria	Actinobac.
			

**Cluster 4 - COG classification L (DNA replication, recombination and repair)**			

**GO terms**	GO:0008168	methyltransferase activity	function
	GO:0008757	S-adenosylmethionine-dependent methyltransferase activity	function
	GO:0009008	DNA-methyltransferase activity	function
	GO:0016741	transferase activity, transferring one-carbon groups	function
			
**Organisms**	Bartonella_tribocorum_CIP_105476	bacteria	Alphaprot.
	Cyanothece_PCC_7424	bacteria	Cyanobac.
	Frankia_EAN1pec	bacteria	Actinobac.
	Orientia_tsutsugamushi_Boryong	bacteria	Alphaprot.
	Orientia_tsutsugamushi_Ikeda	bacteria	Alphaprot.
			

**Cluster 5 - COG classification P (Inorganic ion transport and metabolism)**			

**GO terms**	GO:0006811	ion transport	process
	GO:0006812	cation transport	process
	GO:0015674	di-, tri-valent inorganic cation transport	process
			
**Organisms**	Escherichia_coli_K_12_substr__DH10B	bacteria	Gammaprot.
	Methanosarcina_barkeri_fusaro	archaea	Euryarchaeota
	Ralstonia_metallidurans_CH34	bacteria	Betaprot.
			

**Cluster 6 - COG classification D (Cell division and chromosome partitioning)**			

**GO terms**	GO:0004359	glutaminase activity	function
	GO:0042242	cobyrinic acid a, c-diamide synthase activity	function
			
**Organisms**	Borrelia_burgdorferi_ZS7	bacteria	Spirochaetes
	Halobacterium_sp	archaea	Euryarchaeota
	Methanosarcina_barkeri_fusaro	archaea	Euryarchaeota
	Xanthobacter_autotrophicus_Py2	bacteria	Alphaprot.

The cluster numbering is for local reference only. The COG classification, based on the most represented COG category in each cluster (see Methods), is shown in the heading of each cluster. The table shows GO number, description and ontology for each GO term, and name, kingdom, and group for each species.

#### Cluster 1 - metal ion binding

Cluster 1 includes GO terms involved in copper binding. Copper is a cofactor in a number of proteins. For copper-dependent organisms it is a challenge to keep the amount of copper at a sufficient level and at the same time to avoid a too high intracellular level which may be toxic to the organism [44]. In *Dinoroseobacter shibae *(strain DFL 12) the different proteins in the cluster are identified as a multicopper oxidase type 3 and a merR family transcriptional regulator. For *Mycobacterium gilvum *some of the proteins are cytochrome-c oxidase and copper resistance protein CopC while in *N. hamburgensis *we find proteins identified as cytochrome-c oxidase. The Roseobacter lineage is one of the most abundant groups of bacteria in the oceans, and *D. shibae *was isolated from marine dinoflagellates. *M. gilvum *was isolated from river sediment and *N. hamburgensis *has been isolated from several environments [21]. Copper is a trace metal in the ocean, and the planktonic uptake of this metal may lead to depletion of essential metals in the surface seawater [25]. *D. shibae *are aerobic bacteria able to perform anoxygenic photosynthesis, and are most likely to be found in the ocean surface were access to copper is scarce. The organism encodes proteins in the merR family, a group of metal ion sensing regulators that bind metals and activate the transcription of proteins involved in metal ion detoxification [45]. It is reasonable to hypothesize that the bacterium has retained multiple paralogs involved in copper binding for more efficient uptake and regulation, while the copper resistance protein CopC in *M. gilvum *may enhance the survival of the bacterium under extreme copper stress, as is the case e.g. with the copper resistance protein PcoC in *E. coli *[46]. Cytochrome-c oxidase is a large transmembrane protein involved in the respiratory electron transport chain, and the protein has two copper centres: Cu_A _and Cu_B _[47]. Thus the features of this cluster involve copper handling in one way or another; either as a way to gain access to scarce copper storages or as a way to resist copper poisoning.

#### Cluster 2 - cell motility

Consistent with proteome info in HAMAP [21], all of the organisms in cluster 2 have flagella. In addition to motility, the flagellum plays a key role in gene expression in many bacteria [48]. It could also play a role in other processes like adherence to host cells, host cell invasion, biofilm formation, and protein secretion [48]. For instance, a flagella-minus mutant strain of *Agrobacterium tumefaciens *shows attenuated virulence. It has also been shown that the flagella of Yersinia species functions as a secretion system to secrete a virulence factor [49]. *Xanthomonas oryzae *causes the vascular disease rice bacterial blight disease where the bacteria enter the plant through wounded leaf edges. Studies have shown that the bacteria move towards exudates of susceptible rice plants whereas no chemotaxis occurs towards exudates in resistant rice plants. This implies that chemotaxis play a role in pathogenicity before penetration of bacteria into the rice leaf. It has also been shown that mutation in one of the ORFs in the flagellar operon region in *X. oryzae *leads to weak chemotaxis [49,50]. After the bacteria enter the plant tissue, flagella may no longer be instrumental to virulence.

#### Cluster 3 - glycoside hydrolase

The majority of proteins underlying the GO terms in cluster 3 belong to the glycoside hydrolase family. This family includes among others lysozymes, hyaluronidases, chinitases, esterases, and xylosidases [51]. The enzyme is involved in degradation of cellulose and hemicellulose, in anti-bacterial defence strategies (e.g. lysozyme), and pathogenic mechanisms (e.g. neuraminidase). *Anaerocellum thermophilum *(strain DSM 6725) is known to utilize a variety of polysaccharides like crystalline cellulose and hemicellulose [52], while Streptomycetes are one of the most ubiquitous soil-dwelling bacteria and degrade insoluble remains of other organisms like chitin and lignocellulose [53]. Both *Sodalis glossinidus *(strain morsitans) and the two strains of *E. coli *O157:H7 are members of the family of enterobacteriaceae and are in this cluster represented with proteins involved in degradation of cell wall, e.g. lysozyme. *E. coli *O157:H7 is an enterohaemorrhagic bacterium that causes haemorrhagic colitis, a major threat to human health [54] while *S. glossinidus*, a maternally transmitted endosymbiont, are found to infect a wide range of tissue in tsetse flies - the vector causing sleeping sickness in humans [55]. *S. pyogenes *belong to group A streptococcus (GAS) causing a variety of diseases with a wide range of severity in humans. The bacteria are covered with an outer capsule made of hyaluronic acid to avoid phagocytosis and to facilitate adherence to the epithelial cells [56]. Interestingly, the proteins represented in this cluster are described as hyaluronidases (some of them as phage-associated), an enzyme that degrades hyaluronic acid. The enzyme is lowering the viscosity of hyaluronic acid and thereby increasing the tissue permeability. The hyaluronic acid in the GAS capsule is shown to be structural identical to the mammalian hyaluronic acid, which is a known substrate for GAS hyaluronidase [56]. According to Starr and Engleberg [56], it has not been demonstrated experimentally that the bacterial spread in tissue is facilitated by the hyaluronidase. However, they showed that hyaluronidase may play a nutritional role for the organism under nutrient-starved conditions, making it possible to utilize host hyaluronic acid or its own capsule as a carbon energy source.

#### Cluster 4 - methyltransferase

Cluster 4 is represented with GO terms that involve methyltransferase activity. For *O. tsutsugamushi *(strain Ikeda), the proteins are classified as a N6 adenine-specific DNA-methyltransferase, and for the *O. tsutsugamushi *(strain Boryong and Ikeda) the proteins are described as a site-specific DNA adenine methylase. The genes from *B. tribocorum *are classified as helicase/methyltransferase, phage-related modification methylase and as type III restriction system methylase. In *Cyanothece *(strain PCC 7424) we mainly find various families of transposases while we in *Frankia sp*. (strain EAN1pec) mainly find DNA (cytosine-5) methyltransferase and transposases. Methylation of adenine or cytosine is a part of the restriction modification system in bacteria; by methylating its own DNA by the use of methyltransferases it is possible to separate this DNA from foreign DNA. The foreign DNA will not be methylated in this manner and would then be degraded by specific restriction enzymes. In this way the bacteria is protected from invasions of e.g. bacteriophages. There are certain methyltransferases that are not a part of the restriction modification system, e.g. Dam (DNA adenine methylase) in gammaproteobacteria and CcrM (Cell-cycle regulated Methyltransferase) in alphaproteobacteria. Dam catalyzes the transfer of a methyl group from S-adenosylmethionine to the N6 position of adenine in GATC sequences [40] while CcrM (with a preference for hemi-methylated DNA) methylates the sequence GANTC and has a role in cell-cycle regulation [57]. DNA cytosine methyltransferase which methylates the C-5 position of cytosine in CC(A/T)GG sequences is also not associated with any restriction enzymes [58]. These methyltransferases are part of the regulatory system in a cell, including the control of bacterial virulence; *dam *mutants of *Salmonella enterica, Caenorhabditis elegans, Haemophilus influenzae, Yersinia pseudotuberculosis*, and *Y. pestis *are reported to have attenuated virulence [40,58]. Mutation in *dam *has also shown a reduction on invasion/adhesion to host cells in some, but not all, pathogens [40,57]. Three of the organisms in this cluster belong to the division of alphaproteobacteria where we find the CcrM protein. Adenine methylation is one of the best characterized epigenetic mechanisms for regulation of cell-cycle [59], and a mutation in *ccrM *is found to be lethal in *A. tumefaciens, Brucella abortus, Caulobacter crescentus*, and *Rhizobium meliloti*. Cell-cycle regulation is usually a very tightly controlled process, so in the case of methyltransferase as a cell-cycle regulator it is not clear why the organisms would benefit from more copies of this gene. It is possible that the different copies of methyltransferase may be so-called ecoparalogs; they perform the same function under different conditions, e.g. temperature or pH.

#### Cluster 5 - ion transport

The proteins behind the GO terms in cluster 5 include many iron-enterobactin ABC transporters and nitrate/nitrite transporters in *E. coli *K-12. This strain is found in the lower gut of animals and survives if released to the natural environment [3]. Iron is essential for almost all organisms, but the availability of iron is limited because of low solubility of Fe^3+^. A study by Flo et al [60] has shown that iron is sequestered by Lipocalin 2 as an immune response during infection, which subsequently limits bacterial growth. To acquire iron, the microorganisms release siderophores. Enterobactin is one of the strongest Fe^3+^-binding siderophores that is known today [61]. Duplication of *enterobactin *genes may make it possible for the organism to colonize in poor iron niches like the lower gut. In *Ralstonia metallidurans *(strain CH34) we find proteins described as heavy metal efflux pump, heavy metal resistance proteins, mercuric transport proteins, Hg(II) resistance protein MerP, and bacterioferritin. Heavy metal resistance is one of the main properties to the latter organism and it is therefore often found in sediments and soils with a high content of heavy metals [62]. It has been suggested that the metal resistance is an attribute of multiple layers of efflux pumps with overlapping substrate specificities [63]. In the archaea *Methanosarcina barkeri *(strain fusaro), a nitrogen-fixing organism originally isolated from mud samples but also reported to live in the rumen of cattle [64,65], we find e.g. molybdenum ABC transporters, iron(III) transporters and mercury ion binding proteins. Both molybdenum and iron are essential for most living organisms. Molybdenum is readily available to biological systems and is in fact the most abundant transition metal found in seawater [66]. On the other hand, the availability of Fe^3+ ^is scarce because of limited solubility, as already mentioned. Molybdenum is shown to stimulate the diazotrophic growth in *M. barkeri*, indicating a molybdenum nitrogenase [67]. The duplication of molybdenum ABC transporters may enhance the ability to fixate nitrogen, while the iron(III) ABC transporters make it possible to live where iron availability is low.

#### Cluster 6 - GTPase domain

The protein from *Borrelia burgdorferi *represented in cluster 6 is described as a CobQ/CobB/MinD/ParA nucleotide binding protein, and by comparing this sequence to the COG database by the use of COGnitor, the protein is classified in COG category D and described as "ATPases involved in chromosome partitioning". The proteins from *Halobacterium sp*. (strain NRC-1) are also described as different kinds of chromosome partitioning proteins (sojB, sojC, and sojD), while we in *M. barkeri *and *Xanthobacter autotrophicus *find proteins described as cobyrinic acid a, c-diamide synthase. This latter enzyme catalyzes the conversion of cobyrinic acid to cobyrinic acid a, c-diamide and is involved in the B12-pathway. Cluster 6 is an example of how this type of analysis is sensitive to the quality of sequence annotation. The proteins in this cluster belong to two different groups with respect to the type of process they are involved in; chromosome partitioning and vitamin B12 synthesis. Most of the proteins in the cluster contain a Ras-like GTPase domain, and they are therefore annotated by automatic procedures as functionally related. However, the Ras-like GTPase domain is conserved and widespread in many different proteins [68], and these proteins are not necessarily found in the same pathways. This cluster of paralogs is therefore artificially enlarged by merging together proteins that are not functionally related at a level that is relevant for this analysis.

Even though the mechanisms and the functions of the proteins are not reviewed in full detail in this paper, it is clear that most of the overrepresented GO terms in these clusters can be associated with adaptation to the environment in which the organisms are living.

## Conclusions

We have used statistically overrepresented GO terms associated with duplicated genes to show that these genes often represent features that may give a competitive advantage to the particular organisms when adapting to environmental conditions. We have identified examples of unrelated prokaryotes possibly showing convergent evolution towards a shared environmental niche. We have also investigated functional differences between genes that may and may not handle gene duplication, based on functional COG classification and comparison of paralogs versus singletons. The analysis seems to confirm the hypothesis that paralogs can be associated with adaptive interactions with the environment of the prokaryote. However, the analysis also shows that incomplete or inconsistent annotation as well as ambiguous annotation terms is an important limiting factor regarding automated data analysis.

## Methods

### Data sets

897 complete prokaryotic proteomes were downloaded from NCBI ftp-server [22] in June 2009. For each proteome, a BlastP [69] search against itself was performed and the paralogs were identified using a 75% sequence identity threshold. Maximum number of Blast hits was set to 1000, unless the E-score from Blast exceeded a threshold of 10^-5^. The 200 organisms with the highest paralog fraction, not considering the number of copies of each paralog, were included in the main analysis. To avoid bias because of different genome sizes, the paralog fraction was defined as the ratio between the number of proteins with paralogs and the total number of proteins in each respective organism.

### Graph-representation of similarities between species

Pairwise similarity between species was estimated as the number of common terms in the lists of overrepresented annotation terms from DAVID [26], using all terms (not only GO). In order to reduce the number of random pairwise links at least 10 common terms were required, and the overlap should cover at least 30% of the maximum possible overlap between the two species. The nodes (species) and edges (number of common terms) were loaded into Cytoscape [27], and the map layout was estimated by Cytoscape using the Edge-Weighted Spring Embedded algorithm with the number of common terms as edge weights. Alternative schemes for edge weights were tested, but this had only minor influence on the final result.

### Functional classification of paralogs and clusters of paralogs and species

The paralogs were classified into COGs according to the ptt-files at NCBI. Some paralogs could not be classified, these are registered as "-". Proteins that were classified in two COG categories (e.g. because of multiple domains) were registered in both categories. This paralog classification was used when comparing COG distribution of paralogs and singletons.

The overall COG classification of clusters was based on the same classification as mentioned above, however this classification represents a consensus where the COG category matching the highest number of paralogs in the cluster determines the cluster classification. Additionally, for each of the GO terms included in a cluster we counted the different COG categories for all paralogs included in that GO. The GO term was then assigned a COG category according to the most frequently occurring COG group. The overall functional classification of the cluster was based on all the paralogs in the cluster, not the individual GO term classification. Therefore the overall cluster classification may differ from the COG classification associated with individual GO terms.

### Overrepresentation analysis

The overrepresentation analysis was performed using three different data sets. The paranome from each organism was used as test set in all cases, whereas the three different background sets consisted of all paralogs, all proteomes, and individual proteomes.

The different data sets were analysed by an overrepresentation analysis using DAVID. Only GO terms with a Bonferroni-corrected significance < 0.01 were taken into account, and the identical GO term had to be found in minimum three organisms to be included in the results. The overrepresented GO terms and organisms were clustered with the BiMax algorithm in BicAT version 2.22 [16], using default settings. Clusters for the three data sets were exported from BicAT for further analyses. Only 167 of the initial 200 organisms were included in the exported clusters; 29 of the 33 lacking organisms contained zero or only one GO term, and the remaining four organisms had a unique combination of two GO terms that were not found in other organisms, thus these organisms could not be clustered. All clusters containing only different strains of *E. coli *were removed from further analyses.

### Computing GO similarity scores for clusters using G-SESAME

The GO terms from the clusters were compared against itself with G-SESAME [28] (web server) which computed a similarity score based on the semantic similarity within each cluster. The average GO similarity score for each cluster was computed, and a total average similarity score was computed for each of the three background models. A box and whiskers plot based on the similarity scores was used to compare the different models (Figure 5). The data set with all paralogs as background had the highest average similarity score, implying the most unambiguous clusters, thus this data set was chosen for further analyses.

### Computing phylogenetic distance and verification of Kr-algorithm

The Kr-algorithm version 2.0.2 [42] for alignment-free computation of evolutionary distances was used locally with default settings. FASTA sequences for 16S Ribosomal protein (frn-files) for all organisms were downloaded from the ftp-server at NCBI [22]. The first 16S ribosomal protein in each genome was used. The protein could not be found in 15 of the organisms, for these cases the ribosomal protein sequences were fetched from RDP [43]. A distance matrix for 167 organisms was computed by running the Kr-algorithm. The pre-calculated distance matrices from RDP were our initial choice, but could not be used because some of the organisms in our analyses were not included in this database. To verify that the Kr-algorithm gave evolutionary distances similar to alignment-based estimates we compared it against the RDP distance matrix. Genbank IDs for 137 of the 200 organisms could be mapped and uploaded into RDP. A pre-computed distance matrix based on the 137 organisms was downloaded. The FASTA sequences of 16S ribosomal proteins for the 137 organisms were downloaded and then used as input in Kr and a distance matrix based on this algorithm was computed. The two distance matrices, based on RDP and Kr respectively, are compared in Figure S2 in Additional file 5 and show a good correlation (r = 0.94).

## Authors' contributions

The project was initiated by FD. The overrepresentation analysis with DAVID was performed by BTS, DWH, and RL. Data collection and analysis was done by MSB and JJ. MSB drafted the initial manuscript, and all authors contributed to the final version. All authors have read and approved the final manuscript.

## Supplementary Material

Additional file 1 **The 200 organisms included in analysis**. Table S1 lists all the 200 organisms originally included in the full analysis. Also included are bacterial class, genome size, number of proteins, and paralog fraction. Click here for file

Additional file 2 **Pairwise similarities for clustering of species**. Figure S1 shows distribution of 88404 edges between species representing pairwise similarities. Click here for file

Additional file 3 **Frequency of GO associations**. Table S2 shows the complete results for frequent species - GO term associations. Click here for file

Additional file 4 **Clusters with a high internal similarity and a large phylogenetic distance**. Table S3 shows the clusters from BicAT that have a high internal similarity (computed by G-SESAME) and a large phylogenetic distance (computed by the Kr-algorithm). Also included are COG classifications for these clusters. Click here for file

Additional file 5 **Verification of Kr-algorithm**. Figure S2 shows the correlation between distance matrices from Kr and RDP, computed over 16S ribosomal sequences. Click here for file
