# Supplementary material for: Gene duplications in prokaryotes can be associated with environmental adaptation
Source: BMC Genomics. 2010 Oct 20;11:588. doi: 10.1186/1471-2164-11-588 (PMC3091735; doi:10.1186/1471-2164-11-588)
Supplement: Additional file 1 — The 200 organisms included in analysis. Table S1 lists all the 200 organisms originally included in the full analysis. Also included are bacterial class, genome size, number of proteins, and paralog fraction. [file 1471-2164-11-588-S1.PDF]

Supplementary material

Table S1 The 200 genomes that were included in the analyses performed at DAVID. "A" or "B" in the "Phylum" column represents archaea and bacteria respectively. "# prot" is the total number of proteins in the genome, while "# genes with paralogs" denotes the number of genes that have one or more paralog in the genome (not considering how many paralogs each gene has). "# Total paralogs" are the total number of paralogs in the genome. "Paralog fraction (%)" describes the ratio of #genes with paralogs/#prot. "Total paralog fraction (%)" describes the ratio of #total paralogs/#prot. Not all of the genomes were annotated in DAVID, or the organism did not have enough GO terms in common with the other organisms to be included in results from BicAT, and only 167 organisms were exported from the BicAT analyses. This is indicated with "i" - included or "n" - not included.

Genome	Phylum	Class	Genome size (Mb)	# prot	# genes with paralogs	# total paralogs	Paralog fraction (%)	Total paralog fraction (%)	BicAT
Acaryochloris_marina_MBIC11017	B	Cyanobacteria	8.36	8383	558	1692	6.66	20.18	i
Acidiphilium_cryptum_JF-5	B	Alphaproteobacteria	3.97	3559	118	302	3.32	8.49	i
Acidithiobacillus_ferrooxidans_ATCC_23270	B	Gammaproteobacteria	3	3147	89	199	2.83	6.32	i
Acidithiobacillus_ferrooxidans_ATCC_53993	B	Other Bacteria	2.9	2826	133	343	4.71	12.14	i
Acidovorax_avenae_citrulli_AAC00-1	B	Betaproteobacteria	5.4	4709	108	320	2.29	6.80	i
Acidovorax_sp_JS42	B	Betaproteobacteria	4.54	4155	148	409	3.56	9.84	n
Acinetobacter_baumannii_AB0057	B	Gammaproteobacteria	4.11	3801	94	239	2.47	6.29	i
Alkaliphilus_metalliredigens_QYMF	B	Firmicutes	4.9	4625	162	448	3.50	9.69	i
Anaerocellum_thermophilum_DSM_6725	B	Firmicutes	2.91	2666	68	196	2.55	7.35	i
Anaplasma_marginale_Florida	B	Alphaproteobacteria	1.2	940	24	75	2.55	7.98	n

Anaplasma_marginale_St_Maries	B	Alphaproteobacteria	1.2	948	22	88	2.32	9.28	n
Anaplasma_phagocytophilum_HZ	B	Alphaproteobacteria	1.47	1264	87	228	6.88	18.04	i
Arthrobacter_FB24	B	Actinobacteria	5.08	4506	103	239	2.29	5.30	i
Aster_yellows_witches-broom_phytoplasma_AYWB	B	Actinobacteria	0.73	693	84	289	12.12	41.70	i
Azoarcus_sp_EbN1	B	Betaproteobacteria	4.4	4590	165	627	3.59	13.66	i
Azotobacter_vinelandii_DJ	B	Gammaproteobacteria	5.4	5051	137	391	2.71	7.74	n
Bacteroides_thetaiotaomicron_VPI-5482	B	Bacteroidetes/Chlorobi	6.33	4816	112	293	2.33	6.08	i
Bacteroides_vulgatus_ATCC_8482	B	Bacteroidetes/Chlorobi	5.2	4065	144	346	3.54	8.51	i
Bartonella_bacilliformis_KC583	B	Alphaproteobacteria	1.4	1283	48	103	3.74	8.03	i
Bartonella_henselae_Houston-1	B	Alphaproteobacteria	1.93	1488	68	183	4.57	12.30	i
Bartonella_tribocorum_CIP_105476	B	Alphaproteobacteria	2.62	2092	207	728	9.89	34.80	i
Bifidobacterium_longum_infantis_ATCC_15697	B	Actinobacteria	2.8	2416	62	146	2.57	6.04	i
Bordetella_petrii	B	Betaproteobacteria	5.3	5027	138	456	2.75	9.07	i
Borrelia_afzelii_PKo	B	Spirochaetes	1.24	1214	33	83	2.72	6.84	i
Borrelia_burgdorferi	B	Spirochaetes	1.52	1640	102	422	6.22	25.73	i
Borrelia_burgdorferi_ZS7	B	Spirochaetes	1.35	1239	51	192	4.12	15.50	i
Borrelia_duttonii_Ly	B	Spirochaetes	1.57	1305	75	225	5.75	17.24	n
Borrelia_garinii_PBi	B	Spirochaetes	1.22	1608	81	227	5.04	14.12	i
Burkholderia_cenocepacia_J2315	B	Betaproteobacteria	8.07	7116	165	396	2.32	5.56	i
Burkholderia_glumae_BGR1	B	Betaproteobacteria	7.24	5773	171	507	2.96	8.78	n
Burkholderia_phymatum_STM815	B	Betaproteobacteria	8.7	7496	179	406	2.39	5.42	i
Burkholderia_vietnamiensis_G4	B	Betaproteobacteria	8.4	7617	193	594	2.53	7.80	i
Burkholderia_xenovorans_LB400	B	Betaproteobacteria	9.8	8702	388	882	4.46	10.14	i
Caldicellulosiruptor_saccharolyticus_DSM_8903	B	Other Bacteria	3	2679	93	303	3.47	11.31	i
Campylobacter_jejuni_doylei_269_97	B	Epsilonproteobacteria	1.8	1731	40	86	2.31	4.97	i
Candidatus_Amoebophilus_asiaticus_5a2	B	Bacteroidetes/Chlorobi	1.9	1283	51	179	3.98	13.95	n
Candidatus_Azobacteroides_pseudotrichonymphae_genomovar__CFP2	B	Bacteroidetes/Chlorobi	1.21	852	25	66	2.93	7.75	i
Candidatus_Hamiltonella_defensa_5AT__Acyrthosiphon_pisum	B	Gammaproteobacteria	2.16	2148	139	432	6.47	20.11	n
Candidatus_Methanosphaerula_palustris_E1_9c	A	Euryarchaeota	2.92	2655	65	195	2.45	7.34	i
Candidatus_Phytoplasma_australiense	B	Firmicutes	0.88	684	46	218	6.73	31.87	i
Candidatus_Phytoplasma_mali	B	Other Bacteria	0.6	479	36	78	7.52	16.28	n
Clostridium_beijerinckii_NCIMB_8052	B	Firmicutes	6	5020	127	310	2.53	6.18	i

Clostridium_botulinum_A2_Kyoto	B	Firmicutes	4.2	3878	94	226	2.42	5.83	n
Clostridium_difficile_630	B	Firmicutes	4.31	3753	128	276	3.41	7.35	i
Clostridium_kluyveri_DSM_555	B	Firmicutes	4.06	3913	151	356	3.86	9.10	i
Cyanothece_PCC_7424	B	Cyanobacteria	6.52	5710	159	373	2.78	6.53	i
Dechloromonas_aromatica_RCB	B	Betaproteobacteria	4.5	4171	100	214	2.40	5.13	i
Dehalococcoides_ethenogenes_195	B	Chloroflexi	1.47	1580	63	152	3.99	9.62	i
Delftia_acidovorans_SPH-1	B	Betaproteobacteria	6.8	6040	142	318	2.35	5.26	i
Desulfitobacterium_hafniense_Y51	B	Firmicutes	5.73	5060	139	342	2.75	6.76	i
Desulfobacterium_autotrophicum_HRM2	B	Deltaproteobacteria	5.67	4943	138	380	2.79	7.69	n
Desulfotalea_psychrophila_LSv54	B	Deltaproteobacteria	3.64	3234	76	169	2.35	5.23	i
Desulfotomaculum_reducens_MI-1	B	Firmicutes	3.6	3276	80	239	2.44	7.30	i
Desulfovibrio_desulfuricans_G20	B	Deltaproteobacteria	3.73	3775	108	274	2.86	7.26	i
Desulfovibrio_magneticus_RS_1	B	Deltaproteobacteria	0.07	4700	143	415	3.04	8.83	n
Dinoroseobacter_shibae_DFL_12	B	Alphaproteobacteria	4.43	4187	148	330	3.53	7.88	i
Ehrlichia_chaffeensis_Arkansas	B	Alphaproteobacteria	1.18	1105	38	80	3.44	7.24	i
Ehrlichia_ruminantium_Gardel	B	Alphaproteobacteria	1.5	950	22	47	2.32	4.95	i
Ehrlichia_ruminantium_str._Welgevonden	B	Alphaproteobacteria	1.52	958	27	57	2.82	5.95	i
Enterococcus_faecalis_V583	B	Firmicutes	3.36	3265	84	202	2.57	6.19	i
Escherichia_coli_55989	B	Gammaproteobacteria	5.2	4763	125	371	2.62	7.79	i
Escherichia_coli_APEC_O1	B	Gammaproteobacteria	5.51	4851	124	293	2.56	6.04	i
Escherichia_coli_CFT073	B	Gammaproteobacteria	5.2	5339	161	439	3.02	8.22	i
Escherichia_coli_E24377A	B	Gammaproteobacteria	5.27	4991	131	385	2.62	7.71	i
Escherichia_coli_ED1a	B	Gammaproteobacteria	5.2	4915	157	477	3.19	9.70	i
Escherichia_coli_K_12_substr__DH10B	B	Gammaproteobacteria	4.7	4126	157	367	3.81	8.89	i
Escherichia_coli_O157_H7_EC4115	B	Gammaproteobacteria	5.73	5477	236	803	4.31	14.66	i
Escherichia_coli_O157H7	B	Gammaproteobacteria	5.6	5318	215	698	4.04	13.13	i
Escherichia_coli_O157H7_EDL933	B	Gammaproteobacteria	5.59	5411	288	908	5.32	16.78	i
Escherichia_coli_S88	B	Gammaproteobacteria	5	4696	116	289	2.47	6.15	i
Escherichia_coli_SE11	B	Gammaproteobacteria	5.17	5002	127	317	2.54	6.34	i
Eubacterium_rectale_ATCC_33656	B	Firmicutes	3.4	3621	157	355	4.34	9.80	n
Frankia_CcI3	B	Actinobacteria	5.4	4499	131	363	2.91	8.07	i
Frankia_EAN1pec	B	Actinobacteria	9	7191	334	887	4.64	12.33	i

Geobacter_uraniumreducens_Rf4	B	Deltaproteobacteria	5.1	4357	102	289	2.34	6.63	i
Gluconacetobacter_diazotrophicus_PAl_5	B	Alphaproteobacteria	3.96	3501	90	265	2.57	7.57	n
Gluconacetobacter_diazotrophicus_PAl5	B	Alphaproteobacteria	3.93	3852	113	375	2.93	9.74	i
Halobacterium_salinarum_R1	A	Euryarchaeota	2.66	2749	133	335	4.84	12.19	i
Halobacterium_sp	A	Euryarchaeota	2.57	2622	122	359	4.65	13.69	i
Halorubrum_lacusprofundi_ATCC_49239	A	Euryarchaeota	3.66	3560	88	257	2.47	7.22	i
Helicobacter_acinonychis_Sheeba	B	Epsilonproteobacteria	1.6	1618	43	94	2.66	5.81	i
Klebsiella_pneumoniae_MGH_78578	B	Gammaproteobacteria	5.69	5185	144	334	2.78	6.44	i
Kosmotoga_olearia_TBF_19_5_1	B	Thermotogae	2.3	2118	57	178	2.69	8.40	n
Lactobacillus_delbrueckii_bulgaricus_ATCC_BAA-365	B	Firmicutes	1.9	1721	52	129	3.02	7.50	n
Lactobacillus_gasseri_ATCC_33323	B	Firmicutes	1.9	1755	65	133	3.70	7.58	i
Lactobacillus_reuteri_DSM_20016	B	Firmicutes	2	1900	55	154	2.89	8.11	i
Lactobacillus_reuteri_F275_Kitasato	B	Firmicutes	2	1820	43	123	2.36	6.76	i
Lactococcus_lactis	B	Firmicutes	2.4	2321	67	189	2.89	8.14	i
Lactococcus_lactis_cremoris_MG1363	B	Firmicutes	2.5	2434	57	179	2.34	7.35	n
Lactococcus_lactis_cremoris_SK11	B	Firmicutes	2.56	2504	59	271	2.36	10.82	i
Laribacter_hongkongensis_HLHK9	B	Betaproteobacteria	3.2	3235	96	236	2.97	7.30	n
Magnetococcus_MC-1	B	Other Bacteria	4.7	3716	143	660	3.85	17.76	i
Magnetospirillum_magneticum_AMB-1	B	Alphaproteobacteria	5	4559	140	342	3.07	7.50	i
Marinobacter_aquaeolei_VT8	B	Gammaproteobacteria	4.75	4272	158	417	3.70	9.76	i
Mesorhizobium_loti	B	Alphaproteobacteria	7.6	7272	207	494	2.85	6.79	i
Methanosarcina_acetivorans	A	Euryarchaeota	5.75	4540	209	686	4.60	15.11	i
Methanosarcina_barkeri_fusaro	A	Euryarchaeota	4.84	3624	174	536	4.80	14.79	i
Methanosarcina_mazei	A	Euryarchaeota	4.1	3370	131	353	3.89	10.47	i
Methylibium_petroleiphilum_PM1	B	Betaproteobacteria	4.6	4449	107	255	2.41	5.73	i
Methylobacillus_flagellatus_KT	B	Betaproteobacteria	3	2753	158	321	5.74	11.66	i
Methylobacterium_4_46	B	Alphaproteobacteria	7.78	6692	190	462	2.84	6.90	i
Methylobacterium_chloromethanicum_CM4	B	Alphaproteobacteria	6.2	5516	178	381	3.23	6.91	i
Methylobacterium_extorquens_PA1	B	Alphaproteobacteria	7.78	4829	117	268	2.42	5.55	i
Methylobacterium_nodulans_ORS_2060	B	Alphaproteobacteria	6.2	8308	564	1641	6.79	19.75	i
Methylobacterium_radiotolerans_JCM_2831	B	Alphaproteobacteria	6.92	6431	161	370	2.50	5.75	i
Microcystis_aeruginosa_NIES_843	B	Cyanobacteria	5.8	6312	384	1947	6.08	30.85	i

Mycobacterium_avium_104	B	Actinobacteria	5.5	5120	153	427	2.99	8.34	i
Mycobacterium_bovis_BCG_Pasteur_1173P2	B	Actinobacteria	4.4	3952	147	341	3.72	8.63	i
Mycobacterium_bovis_BCG_Tokyo_172	B	Actinobacteria	4.4	3947	101	268	2.56	6.79	i
Mycobacterium_gilvum_PYR-GCK	B	Actinobacteria	5.96	5579	174	453	3.12	8.12	i
Mycobacterium_KMS	B	Actinobacteria	6.22	5975	179	440	3.00	7.36	i
Mycobacterium_tuberculosis_H37Ra	B	Actinobacteria	4.4	4034	95	262	2.35	6.49	i
Mycobacterium_tuberculosis_H37Rv	B	Actinobacteria	4.4	3989	93	257	2.33	6.44	i
Mycoplasma_gallisepticum	B	Firmicutes	1	726	21	126	2.89	17.36	i
Mycoplasma_hyopneumoniae_232	B	Firmicutes	0.89	691	25	53	3.62	7.67	i
Mycoplasma_hyopneumoniae_7448	B	Firmicutes	0.92	657	16	44	2.44	6.70	i
Mycoplasma_hyopneumoniae_J	B	Firmicutes	0.9	657	19	48	2.89	7.31	n
Mycoplasma_mycoides	B	Firmicutes	1.2	1016	69	228	6.79	22.44	i
Mycoplasma_penetrans	B	Firmicutes	1.36	1037	29	96	2.80	9.26	i
Mycoplasma_pneumoniae	B	Firmicutes	0.82	689	33	106	4.79	15.38	i
Mycoplasma_synoviae_53	B	Firmicutes	0.8	659	35	108	5.31	16.39	n
Natronomonas_pharaonis	A	Euryarchaeota	2.75	2822	69	147	2.45	5.21	i
Neisseria_gonorrhoeae_FA_1090	B	Betaproteobacteria	2.15	2002	56	161	2.80	8.04	i
Neisseria_gonorrhoeae_NCCP11945	B	Betaproteobacteria	2.2	2674	98	337	3.66	12.60	i
Neisseria_meningitidis_053442	B	Betaproteobacteria	2.2	2020	73	233	3.61	11.53	i
Neisseria_meningitidis_FAM18	B	Betaproteobacteria	2.2	1917	47	155	2.45	8.09	i
Neisseria_meningitidis_MC58	B	Betaproteobacteria	2.3	2063	184	475	8.92	23.02	i
Neisseria_meningitidis_Z2491	B	Betaproteobacteria	2.2	1909	44	119	2.30	6.23	i
Nitrobacter_hamburgensis_X14	B	Alphaproteobacteria	5.01	4326	220	565	5.09	13.06	i
Nitrobacter_winogradskyi_Nb-255	B	Alphaproteobacteria	3.4	3122	72	315	2.31	10.09	i
Nitrosomonas_eutropha_C71	B	Betaproteobacteria	2.82	2551	85	288	3.33	11.29	i
Nitrosospira_multiformis_ATCC_25196	B	Betaproteobacteria	3.25	2805	72	215	2.57	7.66	i
Nostoc_punctiforme_PCC_73102	B	Cyanobacteria	9.01	6690	192	546	2.87	8.16	i
Onion_yellows_phytoplasma	B	Firmicutes	0.86	754	58	287	7.69	38.06	i
Orientia_tsutsugamushi_Boryong	B	Alphaproteobacteria	2.1	1182	72	486	6.09	41.12	i
Orientia_tsutsugamushi_Ikeda	B	Alphaproteobacteria	2	1967	151	1690	7.68	85.92	i
Parachlamydia_sp_UWE25	B	Chlamydiae/Verrucomicrobia	2.41	2031	97	262	4.78	12.90	i
Paracoccus_denitrificans_PD1222	B	Alphaproteobacteria	5.25	5077	192	425	3.78	8.37	i

Pelobacter_propionicus_DSM_2379	B	Deltaproteobacteria	4.23	3804	97	276	2.55	7.26	i
Pelotomaculum_thermopropionicum_SI	B	Firmicutes	3	2918	89	249	3.05	8.53	n
Petrotoga_mobilis_SJ95	B	Thermotogae	2.2	1898	44	116	2.32	6.11	i
Photobacterium_profundum_SS9	B	Gammaproteobacteria	6.38	5489	126	576	2.30	10.49	i
Photorhabdus_luminescens	B	Gammaproteobacteria	5.69	4683	207	685	4.42	14.63	i
Pirellula_sp	B	Planctomycetes	7.1	7325	191	574	2.61	7.84	i
Polaromonas_naphthalenivorans_CJ2	B	Betaproteobacteria	5.35	4929	129	341	2.62	6.92	i
Porphyromonas_gingivalis_ATCC_33277	B	Bacteroidetes/Chlorobi	2.4	2090	74	257	3.54	12.30	i
Prochlorococcus_marinus_MIT_9303	B	Cyanobacteria	2.7	2997	81	225	2.70	7.51	n
Ralstonia_eutropha_H16	B	Betaproteobacteria	7.45	6626	178	390	2.69	5.89	i
Ralstonia_metallidurans_CH34	B	Betaproteobacteria	6.91	6319	152	417	2.41	6.60	i
Ralstonia_pickettii_12J	B	Betaproteobacteria	5.28	4952	119	308	2.40	6.22	i
Ralstonia_solanacearum	B	Betaproteobacteria	5.8	5113	155	410	3.03	8.02	i
Rhizobium_NGR234	B	Alphaproteobacteria	6.84	6376	204	543	3.20	8.52	i
Rhodobacter_sphaeroides_ATCC_17025	B	Alphaproteobacteria	4.54	4333	147	466	3.39	10.75	i
Rhodococcus_jostii_RHA1	B	Actinobacteria	9.67	9145	435	1086	4.76	11.88	i
Rhodococcus_opacus_B4	B	Actinobacteria	7.9	7246	169	372	2.33	5.13	n
Rickettsia_felis_URRWXCal2	B	Alphaproteobacteria	1.59	1512	76	231	5.03	15.28	n
Shewanella_baltica_OS155	B	Gammaproteobacteria	5.32	4489	150	521	3.34	11.61	i
Shewanella_baltica_OS195	B	Gammaproteobacteria	5.5	4688	109	338	2.33	7.21	i
Shigella_boydii_CDC_3083_94	B	Gammaproteobacteria	4.86	4557	111	910	2.44	19.97	i
Shigella_flexneri_2a	B	Gammaproteobacteria	4.82	4440	117	846	2.64	19.05	i
Sodalis_glossinidius_morsitans	B	Gammaproteobacteria	4.29	2516	83	239	3.30	9.50	i
Staphylococcus_aureus_JH1	B	Firmicutes	2.93	2780	79	192	2.84	6.91	i
Staphylococcus_aureus_JH9	B	Firmicutes	2.93	2726	77	187	2.82	6.86	i
Staphylococcus_aureus_Newman	B	Firmicutes	2.9	2614	86	228	3.29	8.72	i
Streptococcus_agalactiae_NEM316	B	Firmicutes	2.2	2094	61	173	2.91	8.26	i
Streptococcus_equi_zooepidemicus	B	Firmicutes	2.1	1869	46	136	2.46	7.28	n
Streptococcus_equi_zooepidemicus_MGCS10565	B	Firmicutes	2	1893	44	129	2.32	6.81	i
Streptococcus_pneumoniae_70585	B	Firmicutes	2.2	2202	67	294	3.04	13.35	n
Streptococcus_pneumoniae_CGSP14	B	Firmicutes	2.2	2206	79	264	3.58	11.97	n
Streptococcus_pneumoniae_G54	B	Firmicutes	2.1	2115	52	172	2.46	8.13	n

Streptococcus_pneumoniae_JJA	B	Firmicutes	2.1	2123	55	250	2.59	11.78	n
Streptococcus_pneumoniae_P1031	B	Firmicutes	2.1	2073	52	251	2.51	12.11	n
Streptococcus_pyogenes_MGAS10270	B	Firmicutes	1.9	1986	50	108	2.52	5.44	i
Streptococcus_pyogenes_MGAS315	B	Firmicutes	1.9	1865	52	129	2.79	6.92	i
Streptococcus_pyogenes_MGAS8232	B	Firmicutes	1.9	1839	42	117	2.28	6.36	i
Streptococcus_pyogenes_SSI-1	B	Firmicutes	1.9	1861	44	112	2.36	6.02	i
Streptomyces_coelicolor	B	Actinobacteria	9.09	8154	231	500	2.83	6.13	i
Streptomyces_griseus_NBRC_13350	B	Actinobacteria	8.5	7136	177	395	2.48	5.54	i
Sulfolobus_islandicus_I_G_57_14	A	Crenarchaeota	2.7	2903	73	371	2.51	12.78	n
Sulfolobus_solfataricus	A	Crenarchaeota	3	2977	158	745	5.31	25.03	i
Synechocystis_PCC6803	B	Cyanobacteria	3.94	3569	88	312	2.47	8.74	i
Thauera_MZ1T	B	Betaproteobacteria	4.58	3978	95	217	2.39	5.46	n
Thermoanaerobacter_pseudethanolicus_ATCC_33223	B	Firmicutes	2.4	2243	65	190	2.90	8.47	i
Thermoanaerobacter_tengcongensis	B	Firmicutes	2.69	2588	75	243	2.90	9.39	i
Thermoanaerobacter_X514	B	Firmicutes	2.5	2349	65	155	2.77	6.60	i
Thermodesulfovibrio_yellowstonii_DSM_11347	B	Other Bacteria	2	2033	57	119	2.80	5.85	i
Treponema_denticola_ATCC_35405	B	Spirochaetes	2.8	2767	69	188	2.49	6.79	i
Trichodesmium_erythraeum_IMS101	B	Cyanobacteria	7.8	4451	177	714	3.98	16.04	i
Verminephrobacter_eiseniae_EF01-2	B	Betaproteobacteria	5.63	4947	133	635	2.69	12.84	i
Vibrio_cholerae_O395	B	Gammaproteobacteria	4.1	3875	119	297	3.07	7.66	i
Vibrio_harveyi_ATCC_BAA-1116	B	Gammaproteobacteria	6.09	6040	142	1180	2.35	19.54	i
Wolbachia_endosymbiont_of_Culex_quinquefasciatus_Pel	B	Alphaproteobacteria	1.5	1275	63	246	4.94	19.29	i
Wolbachia_endosymbiont_of_Drosophila_melanogaster	B	Alphaproteobacteria	1.27	1195	81	238	6.78	19.92	i
Wolbachia_wRi	B	Alphaproteobacteria	1.4	1150	68	242	5.91	21.04	n
Xanthobacter_autotrophicus_Py2	B	Alphaproteobacteria	5.62	5035	200	515	3.97	10.23	i
Xanthomonas_oryzae_PXO99A	B	Gammaproteobacteria	5.2	4988	253	1597	5.07	32.02	i
Xylella_fastidiosa	B	Gammaproteobacteria	2.73	2832	146	338	5.16	11.94	i
Xylella_fastidiosa_M12	B	Gammaproteobacteria	2.5	2104	80	189	3.80	8.98	i
Xylella_fastidiosa_M23	B	Gammaproteobacteria	2.54	2201	108	277	4.91	12.59	i
Xylella_fastidiosa_Temecula1	B	Gammaproteobacteria	2.52	2036	86	224	4.22	11.00	i
