# Supplementary material for: Gene duplications in prokaryotes can be associated with environmental adaptation
Source: BMC Genomics. 2010 Oct 20;11:588. doi: 10.1186/1471-2164-11-588 (PMC3091735; doi:10.1186/1471-2164-11-588)
Supplement: Additional file 2 — Pairwise similarities for clustering of species. Figure S1 shows distribution of 88404 edges between species representing pairwise similarities. [file 1471-2164-11-588-S2.PDF]

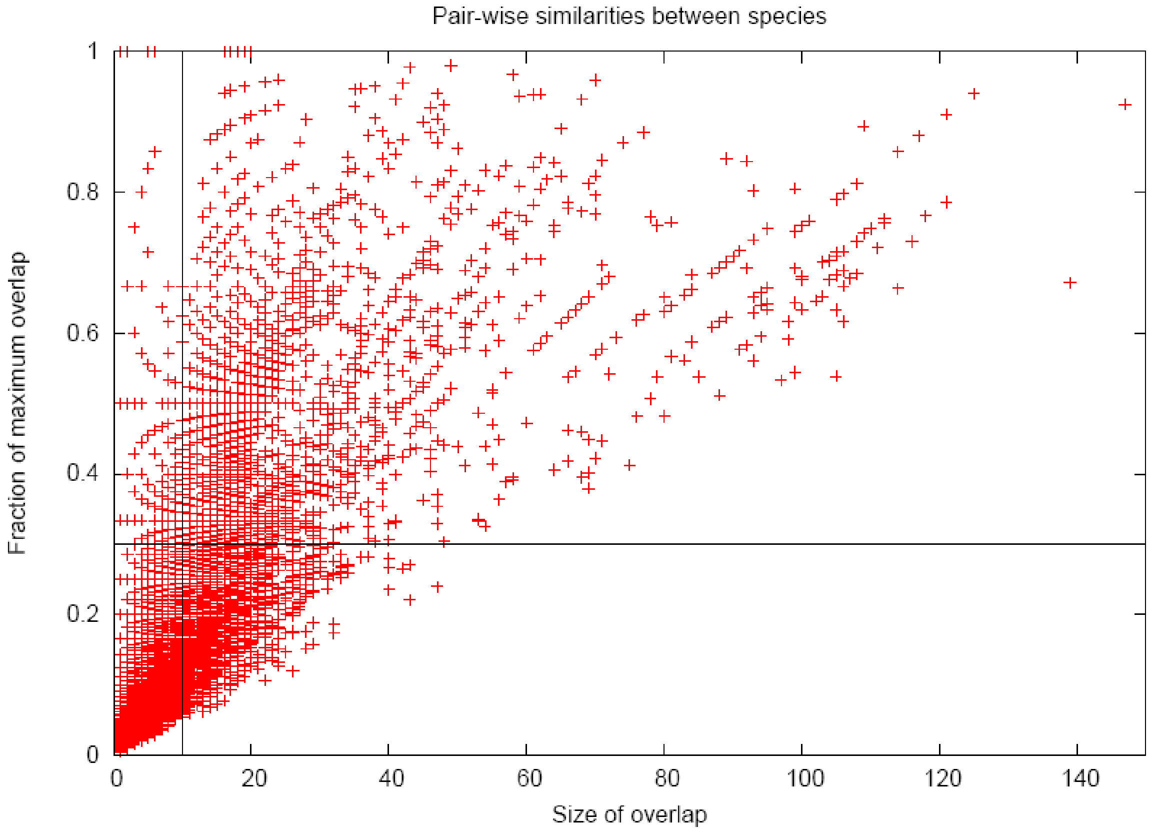

Figure S1 The figure shows distribution of 88404 edges between species representing pairwise similarities. The x-axis shows the size of overlap as number of common annotation terms, the y-axis shows the size of the overlap as fraction of maximum possible overlap between the two species. The lines at x=10 and y=0.3 represents the cut-off values used for generating the graph in Figure 4.
