# Supplementary material for: Gene duplications in prokaryotes can be associated with environmental adaptation
Source: BMC Genomics. 2010 Oct 20;11:588. doi: 10.1186/1471-2164-11-588 (PMC3091735; doi:10.1186/1471-2164-11-588)
Supplement: Additional file 5 — Verification of Kr-algorithm. Figure S2 shows the correlation between distance matrices from Kr and RDP, computed over 16S ribosomal sequences. [file 1471-2164-11-588-S5.PDF]

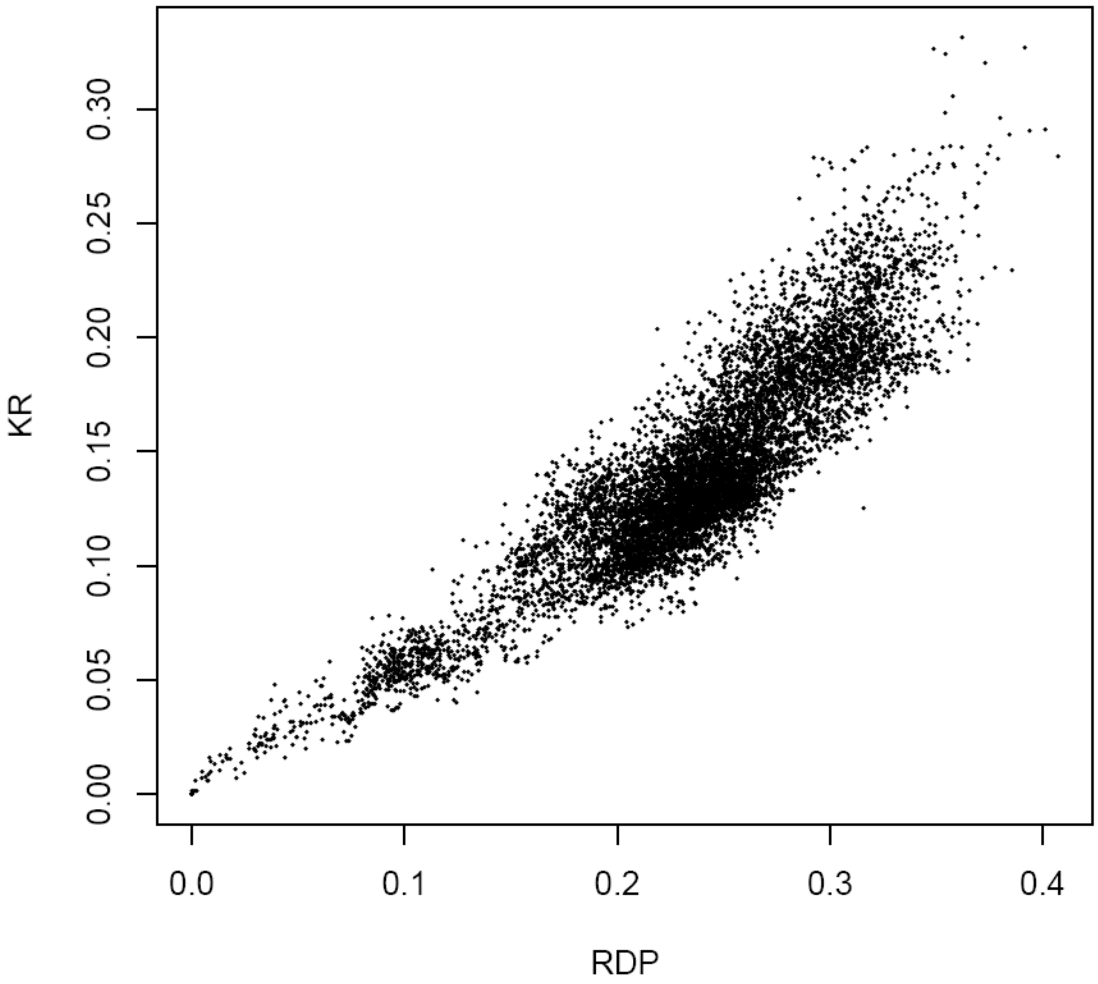

Figure S2 Correlation between distance matrices for 16S ribosomal sequences from Kr and RDP (with Jukes-Cantor correction). 137 genomes are included in the precomputed distance matrix from RDP and the calculated distance matrix from Kr. The figure shows a strong correlation between the distance matrices (r = 0.94), indicating that the alignment-free Kr distance is a reasonable estimate of evolutionary distance.
